# Technical Advances in Skin Sparing Mastectomy

**DOI:** 10.1155/2011/396901

**Published:** 2011-04-28

**Authors:** Grant W. Carlson

**Affiliations:** ^1^Division of Plastic Surgery, Emory University School of Medicine, Atlanta, GA 30322, USA; ^2^Winship Cancer Institute, Emory University, 1365C Clifton Road, Atlanta, GA 30327, USA

## Abstract

Skin sparing mastectomy has resulted in marked improvement in the aesthetic results of immediate breast reconstruction. Mature data has confirmed its oncological safety in the treatment of breast cancer. The procedure has gained wide acceptance and has undergone numerous technical advances since its introduction over twenty years ago. Careful patient selection and choice of skin incisions are necessary to avoid complications.

## 1. Introduction

The term skin sparing mastectomy (SSM) was first used by Toth and Lappert in 1991 [2]. They described preoperative planning of mastectomy incisions in an attempt to maximize skin preservation and facilitate immediate breast reconstruction (IBR). The procedure removes the breast, nipple-areola complex, previous biopsy incisions, and skin overlying superficial tumors [3]. Preservation of the inframammary fold (IMF) and native skin greatly enhances the aesthetic result of breast reconstruction. The operation has been adopted for patients with early breast cancer treated by total mastectomy and immediate reconstruction but has not gained universal acceptance. Most surgeons surveyed agree that the procedure improves the cosmetic results of immediate breast reconstruction [4].

Despite numerous studies that have demonstrated the oncological safety of the procedure compared to traditional total mastectomy, there are still concerns about the oncological safety [1, 5–9]. One international survey of over 1,000 surgeons found that 78% of respondents believed that the current published literature demonstrated that SSM does not result in higher local recurrence rates of breast cancer, 25% did not believe the data [9]. Despite these concerns, the utilization of skin sparing mastectomy continues to increase [10].

## 2. Completeness of Mastectomy

The breast is a modified cutaneous gland or “skin appendage”. It is enclosed between the superficial and deep layers of the superficial fascia of the anterior abdominal wall. The superficial layer is a very delicate but definite structure. Large axial vessels lie deep to this plane and send vertical branches to the subdermal plexus. This layer allows the surgeon to dissect the skin flaps in a relatively avascular plane and include minimal mammary tissue. Cooper's “ligaments” are peripheral projections of breast tissue in fibrous processes, which fuse with the superficial layer of the superficial fascia [11]. Skiles demonstrated that these projections were intimately associated with the skin and concluded in order to excise the whole breast that a large amount of skin need be sacrificed or the dissection kept as close to it as run a risk of skin slough [12].

Beer et al. examined breast tissue from 62 reduction specimens for the presence of the superficial fascia [13]. They found the superficial fascia was absent in 44% of the inferior breast quadrants examined. When it was present, no breast tissue was found beyond it. Torresan et al. studied the skin that would have been preserved in SSM in 42 mastectomy specimens [14]. They found that the presence of breast tissue was significantly associated with skin flaps thicker than 5 mm. They found no correlation between the presence of breast tissue in the skin flaps and age, body mass index, or menopausal status. Hicken outlined the extent of mammary tissue in 1940 by injecting X-ray contrast material into the lactiferous ducts of 385 mastectomy specimens [15]. He found that in 95% of cases the ducts ascended into the axilla, 15% passed into the epigastric space, and 2% followed the lateral chest wall beyond the anterior border of the latissimus dorsi muscle. This study defined the classic boundaries of a mastectomy: the clavicle, rectus sheath, midline of the sternum, and the anterior border of the latissimus dorsi muscle.

The fascial relationships of the breast facilitate its removal along defined tissue planes. The inferior extent to breast tissue, except in rare cases, stops at the separation of the superficial and deep layers of the superficial fascia of the abdominal wall. Cooper stated that at the “… abdominal margin, the gland is turned upon itself at its edge, and forms a kind of hem” [11]. The zone of adherence of the superficial fascial to the underlying chest wall in this region is the inframammary fold [16]. It occurs at the inferior margin of the pectoralis major muscle at the 6th and occasionally the 7th rib. Its presence has been demonstrated in the 8th month fetus, and its location is fixed throughout life [17]. It generally contains fat, which may become firm and indurated in patients with large, ptotic breasts. Breast cancer is extremely rare in this location. Haagenson, in his large experience, cited only 26 cases of breast cancer occurring in the region of the IMF [18]. Preservation of the inframammary fold leaves minimal amount of breast tissue and does not appreciably effect the completeness of a mastectomy [19].

All forms of mastectomy leave residual breast tissue. The differences between the various mastectomy techniques are in terms of the amount of microscopic breast tissue left behind in the skin. These small differences have not been shown to impact the local recurrence of breast cancer [1, 5–8].

## 3. Types of Skin Sparing Mastectomy

**Figure 1:**
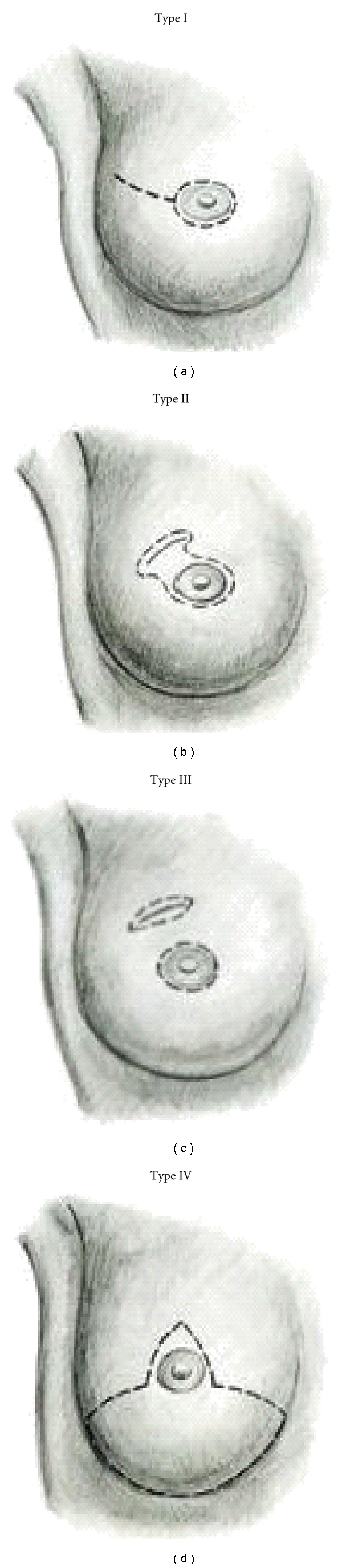
Types of skin sparing mastectomy [1].

**Figure 2:**
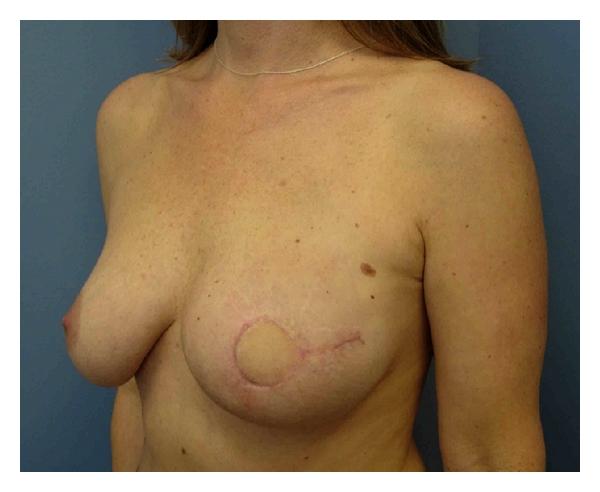
Type I SSM Tennis Racquet Incision.

The type of skin sparing mastectomy has been classified by the type of incision used and the amount of skin removed (Figure 1) [1]. Factors influencing incision choice include previous biopsies, tumor location and depth, and the type of reconstruction planned. A periareolar incision or *Type I SSM* is commonly used in prophylactic cases and for nonpalpable cancers diagnosed by needle biopsy. In patients with small diameter areola, a lateral extension or “tennis racquet incision” is sometimes necessary to improve exposure to the axillary tail, or to provide access for breast reconstruction (Figure 2). If implant/expander reconstruction is planned, the circular incision can be converted to an “elliptical incision”. The incision should be obliquely oriented toward the axilla to reduce flattening of the central breast mound. A purse string closure of the circumareolar incision has been described in patients with small to medium sized breasts undergoing immediate implant reconstruction [20]. In the author's opinion, the closure is slow to heal and the resultant scar can make nipple reconstruction difficult.

**Figure 3:**
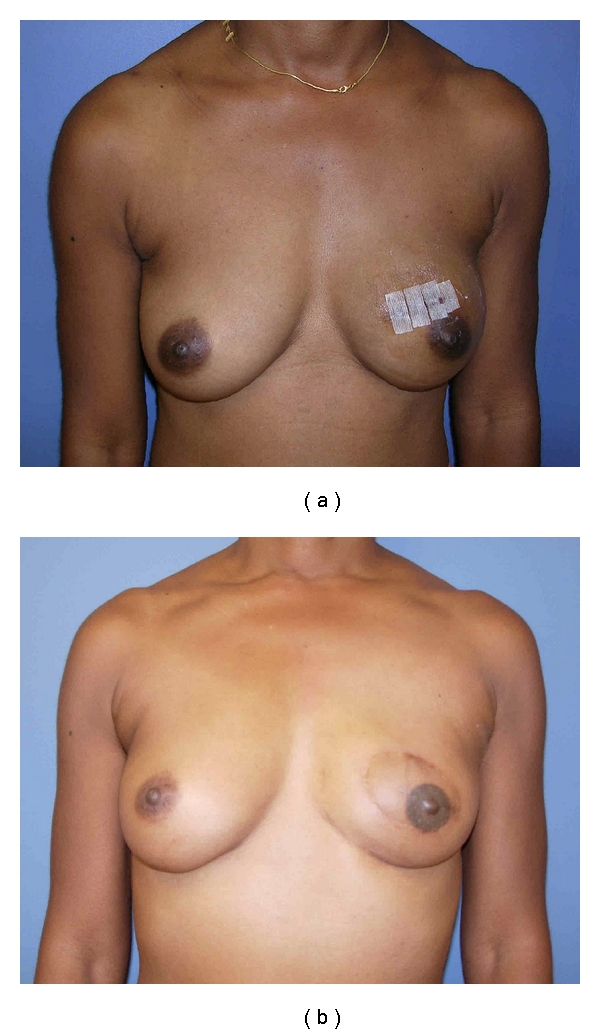
Type II SSM and TRAM Flap Reconstruction.

Previous incisions impact the amount of skin preservation in SSM. The wide adoption of imaging directed stereotaxic core biopsy has reduced the number of excisional breast biopsies. Today, excisional biopsy incisions usually result from failed attempts at breast conservation. The ablative surgeon must be aware of the depth and extent of tumor involvement prior to final decision planning. A *Type II SSM* is used when a superficial tumor or previous biopsy is in proximity to the areola. In autologous reconstruction, the flap skin can be used to fill the defect (Figure 3). In implant-based reconstruction, the skin is closed to facilitate breast shape. *Type III SSM *is used when the superficial tumor or previous incision was remote from the areola, usually in the upper quadrants of the breast. Care must be taken to ensure the viability of the intervening skin. 

**Figure 4:**
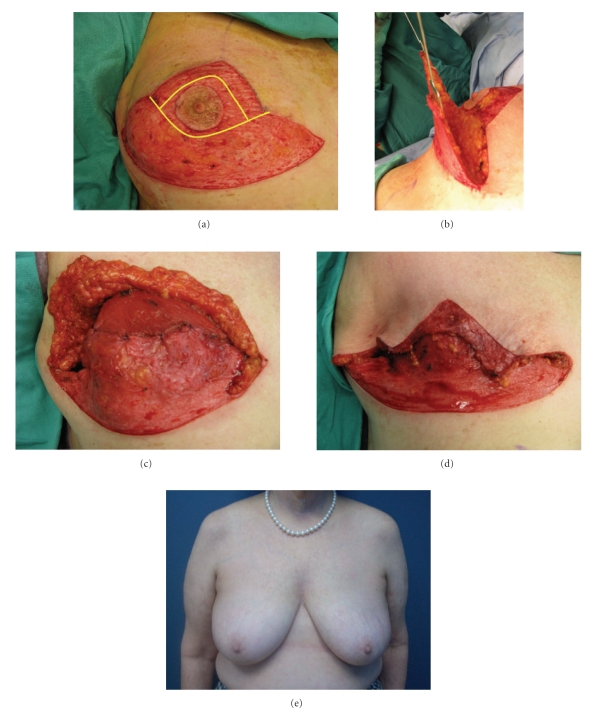
(a) Intraoperative photograph showing Wise pattern incisions with de-epithelialization of the skin. The area in yellow will be resected as part of the Type IV SSM. This excision leaves a rim of dermis along the vertical limbs of the skin excision. (b) The inferior skin flap is elevated down to the inframammary fold. (c) The de-epithelialized inferior skin flap is draped over the tissue expander and sutured to the released inferior border of the pectoralis major muscle. Back cuts are made to allow inset of the dermal flap. (d) The skin flaps just prior to closure. The de-epithelialized vertical limbs serve as a buttress. (e) Preoperative appearance. (f) Postoperative appearance after type IV SSM and implant reconstruction using an inferior dermal flap.

A *Type IV SSM* is used in large, ptotic breasts when a reduction is planned on the opposite breast. A common problem with this technique is the occurrence of native skin flap necrosis of the most distal portions of the flap, particularly at the “T” junction. Skoll has described a modification of the Wise pattern to avoid this complication [21]. The area between the vertical limbs of the T and an additional 2cm outside the horizontal limbs are deepithelialized but not resected. Bostwick first described using Wise pattern mastectomy incisions for prophylactic mastectomies and immediate implant reconstruction [22]. He released the pectoralis major muscle and used a deepithelialized inferior skin flap to allow placement of definitive silicone prosthesis. Other authors have built on Bostwick's work, using an inferior dermal pedicle to cover the prosthesis with acceptable complication rates (Table 1). The technique can obviate the need for an acellular dermal matrix and provide a dermal buttress to reduce wound healing complications (Figure 4).

## 4. Skin Flap Elevation

The skin flaps are elevated superficial to the enveloping fascia of the breast. The skin flap thickness depends on the location on the breast and body habitus of the patient. Breast tissue extends closer to the skin in the lower quadrants and the subcutaneous tissue is thicker in the upper, outer quadrant of the breast. The skin flaps generally are two to five mm. in thickness. Exposure of the white dermis indicates the plane is too superficial to assure skin viability. Electrical cautery on low blended coagulation current is preferred by many surgeons for flap elevation because it is quicker with less blood loss. Care must be taken to avoid thermal injury to the skin. The majority of the blood vessels lie deep to the fascia, but perforating vessels to the skin are encountered and controlled with coagulation current. The infiltration of the breast with dilute epinephrine solution has been described to facilitate sharp dissection of the skin flaps and reduce blood loss [23]. 

**Figure 5:**
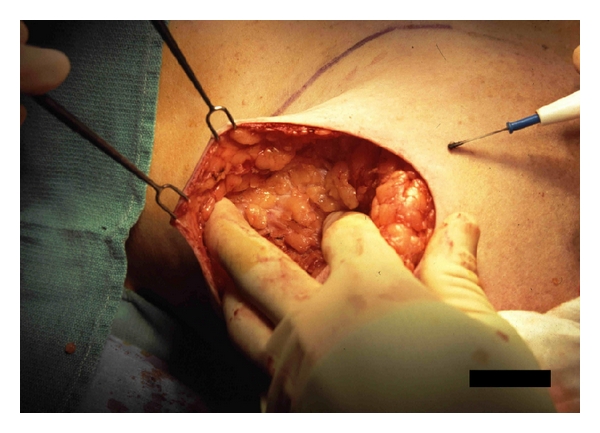
Scissoring of the fingers can assist in deep dissection obviating the need for deep retractors.

Skin retraction is performed with double pronged skin hooks. The flaps must be handled carefully, and the use of deep abdominal retractors is avoided. Scissoring of the fingers of the nondominant hand can assist in the deep dissection (Figure 5). Because the skin opening is small, the flaps are elevated in a centripetal fashion to assist in exposure. Skin flap viability is usually assessed on clinical grounds but the use of epinephrine solution and blue dye that is used in sentinel lymph node mapping may make this difficult. Fluoroscein dye may be helpful in select cases, especially those where implant reconstruction is used, but it tends to underestimate skin viability [24]. Intraoperative indocyanine green angiography has been shown to have a high sensitivity and specificity in predicting native skin flap necrosis after SSM [25].

Superiorly, the breast falls away from the skin as the clavicle is approached. The fascia is followed down to the pectoralis major muscle. Medially, the fascia is not as defined and the dissection ends at the border of the sternum. Perforating vessels of the internal mammary artery are frequently encountered along the sternal border and can be controlled with the cautery. Attempts should be made to preserve these vessels in cases using type IV SSM to improve skin flap blood supply. These perforators can be used as donor vessels in free flap breast reconstruction. Inferiorly, the dissection follows the superficial layer of the fascia to its junction with the deep layer (Figure 4). The skin is adherent to the anterior abdominal wall at this juncture. This fascial junction occurs at the inferior edge of the pectoralis major muscle. Laterally, the dissection continues over the pectoralis muscle toward the humerus enabling removal of the axillary tail. If an axillary incision is required, a tunnel is developed between it and the chest incision. The breast is mobilized laterally over the serratus anterior muscle. The axillary dissection is performed in continuity with the breast tail and the specimen is removed en bloc through the central incision.

## 5. Native Skin Flap Necrosis

The reported incidence of native skin flap necrosis after SSM has been reported to be 10% to 22% when followed by IBR [1, 26, 27]. It can vary in severity from superficial epidermolysis to full thickness skin loss. Predisposing factors include: preoperative radiation, tobacco smoking, incision type, obesity, breast size, and age [28–31]. Skin flap elevation with the scalpel or cautery appears to have similar skin necrosis rates [30]. There are conflicting reports as to its impact of epinephrine infiltration on mastectomy flap viability [29, 30]. A review of the Emory University experience found that Type III and IV incisions, tobacco smoking, and preoperative radiation predisposed to native skin flap necrosis (Table 2) [28]. Davies et al. found that periareolar incisions had a significantly lower rates of complications compared to Wise pattern or tennis racquet incisions [30]. 

**Figure 6:**
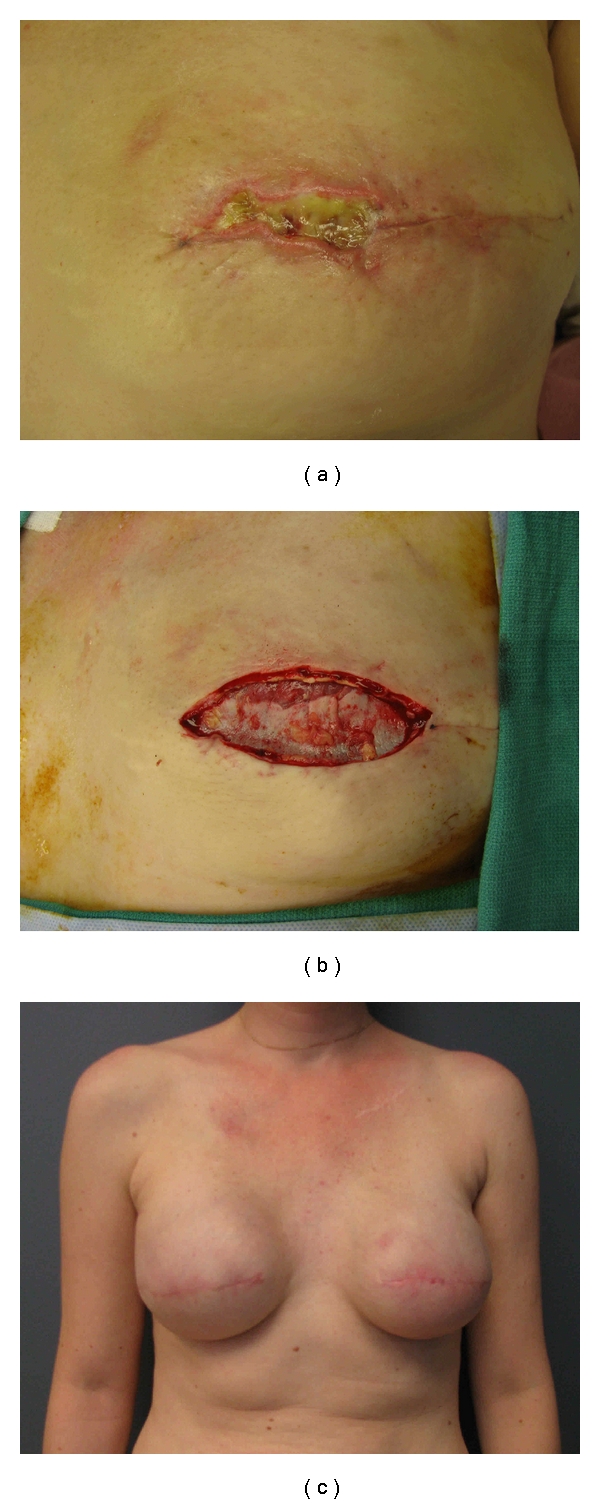
((a), (b)) After excision of the necrotic tissue, the pectoralis major interface with acellular dermal matrix is exposed. (c) Appearance after expansion and placement of permanent implants.

The management of native skin flap necrosis depends on its depth and extent and the type of reconstruction performed. Skin necrosis after expander reconstruction should be managed aggressively to prevent exposure and the development of infection necessitating implant removal (Figure 6). Antony et al. reported their experience with 58 cases of mastectomy skin flap necrosis following tissue expander reconstruction [32]. Nine patients required removal of the expander because of the large area of skin loss. The remaining patients underwent skin excision and primary closure. Three of these patients (6%) subsequently developed infection necessitating expander removal.

## 6. Summary

The use of skin sparing mastectomy has been one of the greatest advancements in immediate breast reconstruction in the last two decades. It is technically more challenging than traditional mastectomy and requires close coordination between the oncologic and reconstructive surgeons. Proper patient selection and meticulous technique are necessary to avoid wound complications.

## Figures and Tables

**Table 1:** Series of Type IV skin sparing mastectomy using an inferior dermal flap.

Author	*N*	Type of reconstruction	Native skin necrosis (%)	Implant removal (%)
Hammond et al. [33]	12	Expander	8	8
Nava et al. [34]	30	Implant	13	13
Derderian et al. [35]	20	Expander/ADM	25	0
Losken et al. [36]	34	Expander	15	12

ADM acellular dermal matrix.

**Table 2:** Risk factors for native skin flap necrosis after SSM [1].

Factor	Total *N* (%)	Native skin flap necrosis *N* (%)
SSM Type		
Type I	232 (36.7)	22 (9.5)
Type II	293 (46.2)	28 (13)
Type III	40 (6.3)	10 (25)
Type IV	68 (10.8)	18 (26.5)
Tobacco	79 (12.5)	16 (20.3)
Radiation	21 (3.3)	5 (23.8)
**Overall**	**633 (100)**	**88 (13.9)**
